# Tracking the Presence of Software as a Medical Device in US Food and Drug Administration Databases: Retrospective Data Analysis

**DOI:** 10.2196/20652

**Published:** 2021-11-03

**Authors:** Aaron Ceross, Jeroen Bergmann

**Affiliations:** 1 Natural Interaction Lab Department of Engineering Science University of Oxford Oxford United Kingdom

**Keywords:** regulation, software, medical device

## Abstract

**Background:**

Software as a medical device (SaMD) has gained the attention of medical device regulatory bodies as the prospects of standalone software for use in diagnositic and therapeutic settings have increased. However, to date, figures related to SaMD have not been made available by regulators, which limits the understanding of how prevalent these devices are and what actions should be taken to regulate them.

**Objective:**

The aim of this study is to empirically evaluate the market approvals and clearances related to SaMD and identify adverse incidents related to these devices.

**Methods:**

Using databases managed by the US medical device regulator, the US Food and Drug Administration (FDA), we identified the counts of SaMD registered with the FDA since 2016 through the use of product codes, mapped the path SaMD takes toward classification, and recorded adverse events.

**Results:**

SaMD does not seem to be registered at a rate dissimilar to that of other medical devices; thus, adverse events for SaMD only comprise a small portion of the total reported number.

**Conclusions:**

Although SaMD has been identified in the literature as an area of development, our analysis suggests that this growth has been modest. These devices are overwhelmingly classified as moderate to high risk, and they take a very particular path to that classification. The digital revolution in health care is less pronounced when evidence related to SaMD is considered. In general, the addition of SaMD to the medical device market seems to mimic that of other medical devices.

## Introduction

### Background

Appropriate application of new digital technologies for health care is dependent on ever-evolving ethical and regulatory frameworks [1]. The US Food and Drug Administration (FDA) defines and oversees this framework for all medical devices in the United States, including software as a medical device (SaMD). Software is an integral part of many health care solutions, and in recent years, it has been acknowledged as a medical device on its own. The International Medical Device Regulators Forum (IMDRF) characterizes software as a medical device when the software itself is considered a medical device without the need for accompanying hardware. The development of more standalone software for clinical applications has led to the recognition of SaMD within medical device regulation. The FDA itself has acknowledged the strong growth potential and development of SaMD [2].

In 2011, the FDA undertook a study to predict trends for medical devices for the next 10 years. The study involved technical managers from the FDA and 15 non-FDA participants with a range of backgrounds, including clinical, policy making, and technological [3]. It was found that reliance on software was a concept that crossed all six identified areas of growth. It was readily accepted that software would not just be an area of growth but also constituted a fundamental component of other trends. Within the last decade, standalone software has increasingly automated and facilitated a range of processes within the medical profession [4]. It has been suggested that industry initiatives around this domain are continuously growing [5], which seems to be in line with popular opinion. However, there has been little empirical work describing the how the available data represent real growth and impact. It is useful to know how quickly this revolution is entering health care and potentially understand any documented issues. In this work, we explore data provided by the FDA to (1) discern new medical device product additions related to software, particularly in relation to SaMD, and (2) identify adverse incidents related to these registered devices. We aim to uncover any patterns in the data that may suggest the nature of any growth of SaMD.

### The FDA Classification Process

Although it may be possible to use an arbitrary device within a clinical setting or to address a medical issue, not all such devices may be categorized as “medical devices” for the purposes of regulation. Section 201(h) of the Food, Drug, and Cosmetic Act [6] provides a definition of a “medical device,” which may be:

any instrument, apparatus, implement, machine, appliance, implant, reagent for in vitro use, software, material or other similar or related article, intended by the manufacturer to be used alone or in combination, for human beings, for one or more [. . . ] specified medical purpose(s) . . .

The “specified medical purposes” covers a wide range of activities, including (1) the diagnosis, prevention, monitoring, treatment, or alleviation of disease or injury; (2) the investigation, replacement, modification, or support of the anatomy or of a physiological process; (3) supporting or sustaining life; (4) the control of conception; or (5) providing information by means of in vitro examination of specimens derived from the human body.

A registered medical device receives a classification according to the risk it poses to the individual. The device’s intended use and purpose provide an indication of the risk level and, thus, the classification. The FDA describes three risk levels that set the types of controls and assessments that need to be considered before the device can be placed on the market [7]. The classification and associated risks and controls are provided in Table 1.

**Table 1:** A generalization of the US Food and Drug Administration risk classifications for medical devices.

Class	Risk level	Controls
Class I	Low to moderate	General controls
Class II	Moderate to high	General controls and special controls
Class III	High	General controls, special controls, premarket clearance

**Figure 1:**
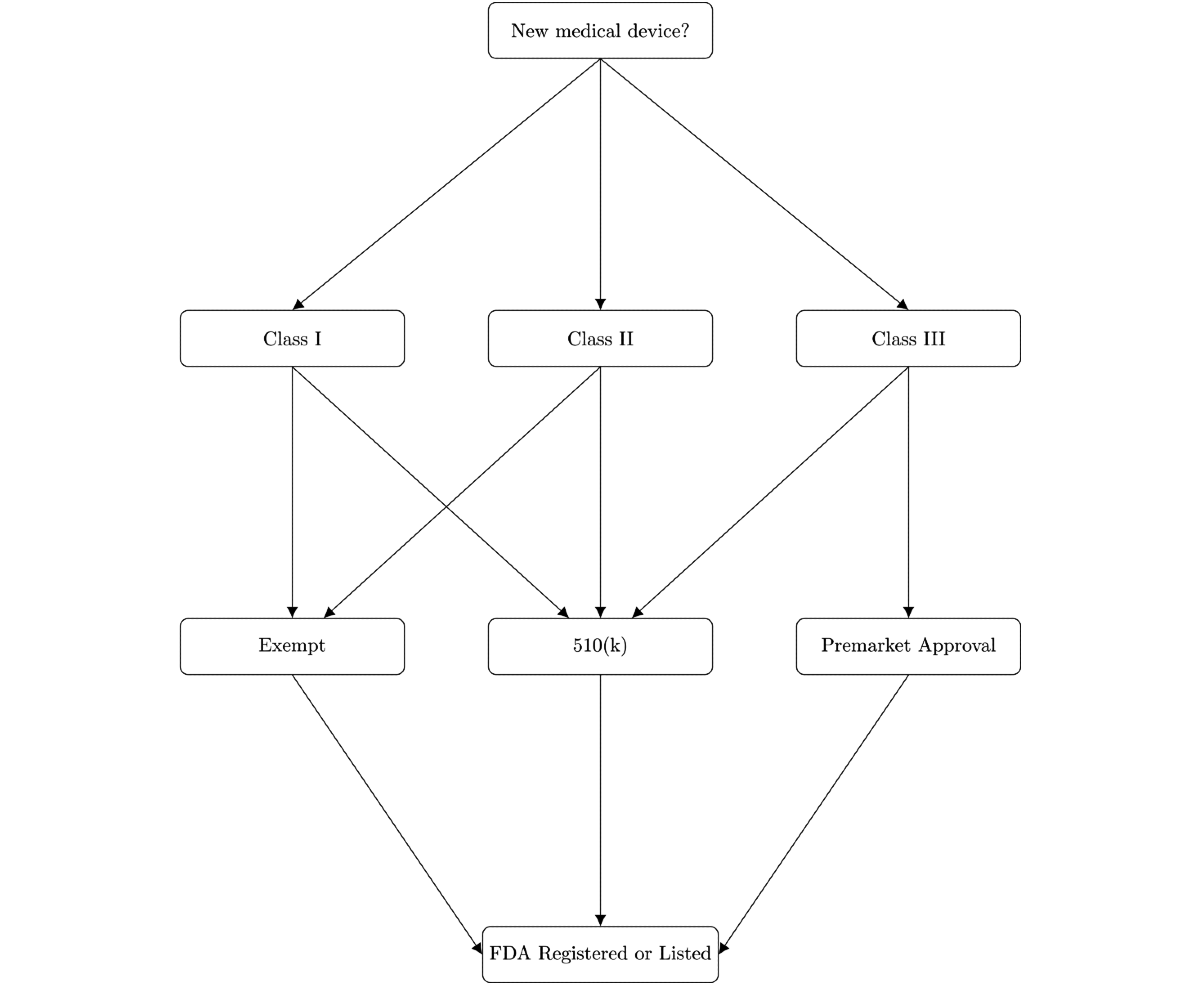
A simplified model of the FDA classification pathways for new medical devices. The 510(k) is a submission made to the FDA to demonstrate that the device to be marketed is equivalent in safety and effectiveness to a legally marketed device, which is not subject to premarket approval. FDA: US Food and Drug Administration.

The classification of a device also plays a role in determining its pathway to final approval as a medical device. Class I devices are exempt from premarket submission. All devices are subject to general controls, which include notification, basic safety measures, and registration requirements. Special controls are those that consist of more stringent risk management processes, such as mandatory reporting of adverse events. Class III devices also require these measures, as well as a premarket approval (PMA) application, due to their high-risk nature (eg, supporting or sustaining human life) or novelty. A PMA involves more rigorous testing of the product before it can be released to market. Figure 1 provides a simplified view of the FDA paths for approval across different classifications.

The FDA uses a system of product codes to facilitate the classification of medical devices. A product code is a three-letter combination that designates the technological type of a device and its class. The Center for Devices and Radiological Health provides the names and attributes of each product code. The classification codes allow for rapid identification of the device type and the types of regulation that would apply, with the aim of making the path to market more efficient and rapid. The 510(k) is a submission made to the FDA to demonstrate that the device to be marketed is equivalent in safety and effectiveness to a legally marketed device, which is not subject to premarket approval.

### SaMD Regulation

In 2013, there was regulatory recognition that standalone software may constitute a medical device, given the proliferation of developed systems. The software was previously classified according to an affiliated hardware device before the explicit inclusion of software in the regulations [8]. The IMDRF is a voluntary group of medical device regulators from different jurisdictions, working together under the World Health Organization’s Global Harmonization Task Force with a focus on the harmonization of medical device regulation. They drafted a guidance document [9] for SaMD to harmonize their definitions. In the document, software was recognized as a device without the need for affiliated hardware. This widened the scope of medical devices to include analytical software as well as mobile apps.

## Methods

### Addition of New Devices

In this work, we used the Global Unique Device Identification Database (GUDID), which is maintained by the FDA and made freely available to the public [10]. It contains records of devices that have a unique identification number. The device companies submit the relevant information concerning their product to the database. The data are made available either through an API or as downloadable text files. GUDID divides its files into 9 separate files [11]. The devices are identified in each of the data sets through a “Primary Device Identifier” number, which is unique to each device. In this work, we used the text files available from the website (full release dated August 21, 2020) and analyzed the data in the R language. The files include the unique ID of the device, description of the device, manufacturer, date of addition to the database, product code, and device classification. The device publish date is the date that the device record was created in the database.

To identify SaMD, we used the Global Medical Device Nomenclature (GMDN) terms [12] and the FDA product codes [13]. The GMDN is an internationally accepted scheme that identifies medical devices through a 5-digit numeric value and generic terms associated with this unique value [9]. In a similar manner, the FDA has developed product codes for medical devices that associate a device with a generic description and type. We extracted SaMDs by subsetting those devices with the string “software” as a term.

We analyzed the data between February 2014 and August 2020, comprising 2,628,409 devices. A total of 32 product codes contain the term “software” in their name for these years. Devices that are software but were not assigned one of the relevant codes previously mentioned were not considered in this analysis. In this work, we used a Sankey diagram to explore and visualize the pathway to market approval. The diagram shows the transitions and relations within the data, allowing for a more immediate understanding of the relationship of the variables within the data.

### Adverse Events

Adverse events related to medical devices are recorded in the Manufacturer and User Facility Device Experience (MAUDE) database [14]. The database comprises both mandatory reports (eg, those obtained from manufacturers) and voluntary reports (received from patients and clinicians). The data are made available as text files in a pipe-delimited format and contain fields related to the product, its name, its type, and the number of affected patients (no personal data are available in these data). These are split into separate files. In this work, we used the device data files. The database acknowledges that it is limited in its surveillance and contains incomplete and/or inaccurate descriptions of events. As such, it is not possible to use MAUDE to detect the prevalence of any type of event, owing to the potential of underreporting. Nevertheless, the data can be informative and provide an indication about the types of risks encountered by patients in the use of medical devices. In addition, we cross-referenced GUDID data information with FDA reports of adverse events through the use of product codes.

## Results

### Addition of New Devices

During the period from 2014 to 2020, 6193 devices were registered with “software” as a GMDN term. However, it is unclear from the GMDN whether the device is solely composed of software or merely incorporates it. To resolve this, we relied on product codes. Of the total devices registered with software product codes, 515 had only a single product code that was related to software. These devices were identified as SaMD. Table 2 shows that most of these devices were Class II, and nearly all that were identified as SaMD by product code (476/515, 92.4%) fell within this classification. It should be noted that the figures for software do not add up to 100% owing to rounding as well as to removal of records listed with unknown device classes.

**Table 2:** Classification proportions for all the GUDID data for software (generally, as a subset by GMDN terms) and SaMD (defined as a subset by product codes).

Class type		Value, n (%)
**Total GUDID^a^ (N=2,628,409)**		
	Class I	599,277 (22.8)
	Class II	1,968,678 (74.9)
	Class III	49,940 (1.9)
**Software GMDN^b^ (n=6193)**		
	Class I	793 (12.8)
	Class II	5208 (84.1)
	Class III	155 (2.5)
**SaMD^c^ product code (n=515)**		
	Class I	12 (2.3)
	Class II	476 (92.4)
	Class III	0 (0)

^a^GUDID: Global Unique Device Identification Database.

^b^GMDN: Global Medical Device Nomenclature.

^c^SaMD: software as a medical device.

**Figure 2:**
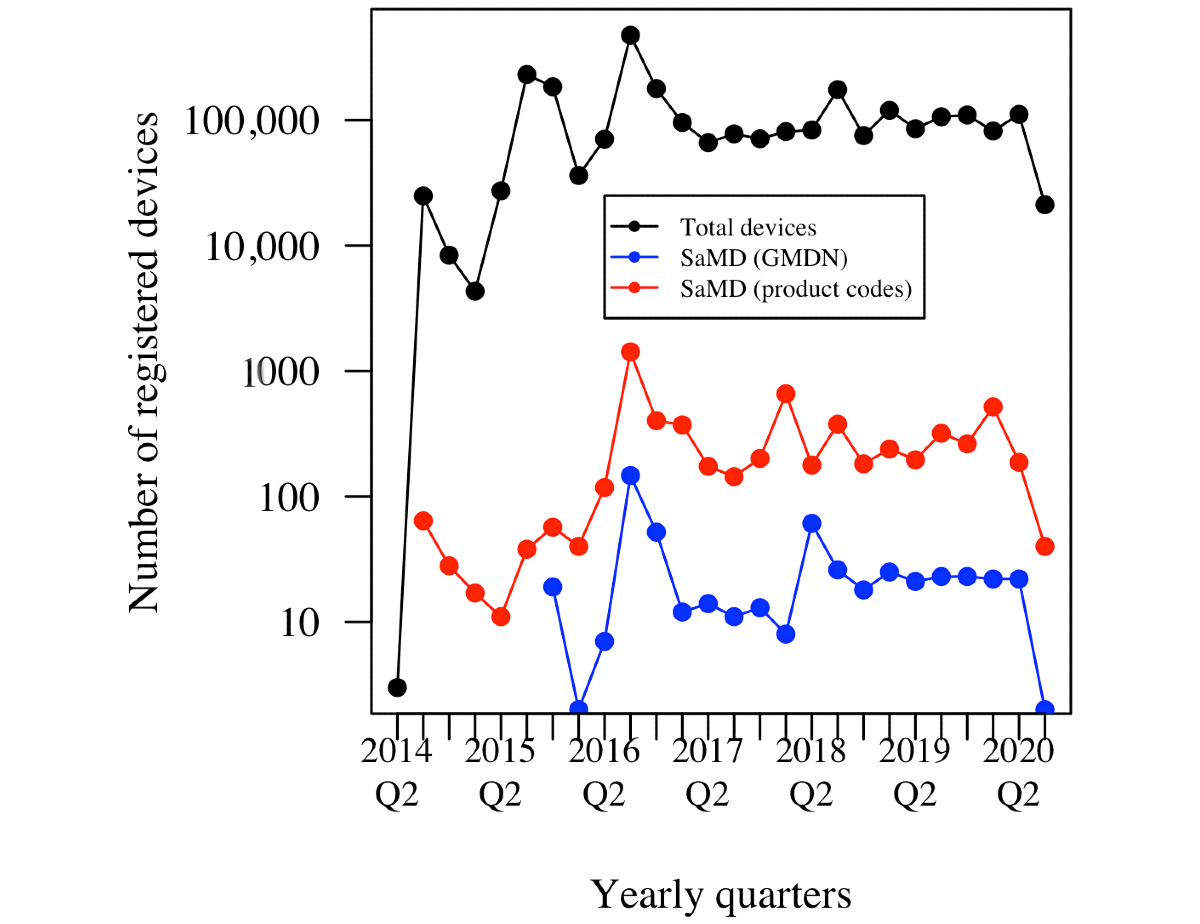
Registration of devices in the Global Unique Device Identification Database by yearly quarter. The vertical axis is on a logarithmic scale. GMDN: Global Medical Device Nomenclature; Q: quarter; SaMD: software as a medical device.

In Figure 2, the patterns of new approvals for software (subset by both GMDN and product codes) and the general pattern for medical devices are shown.

**Figure 3:**
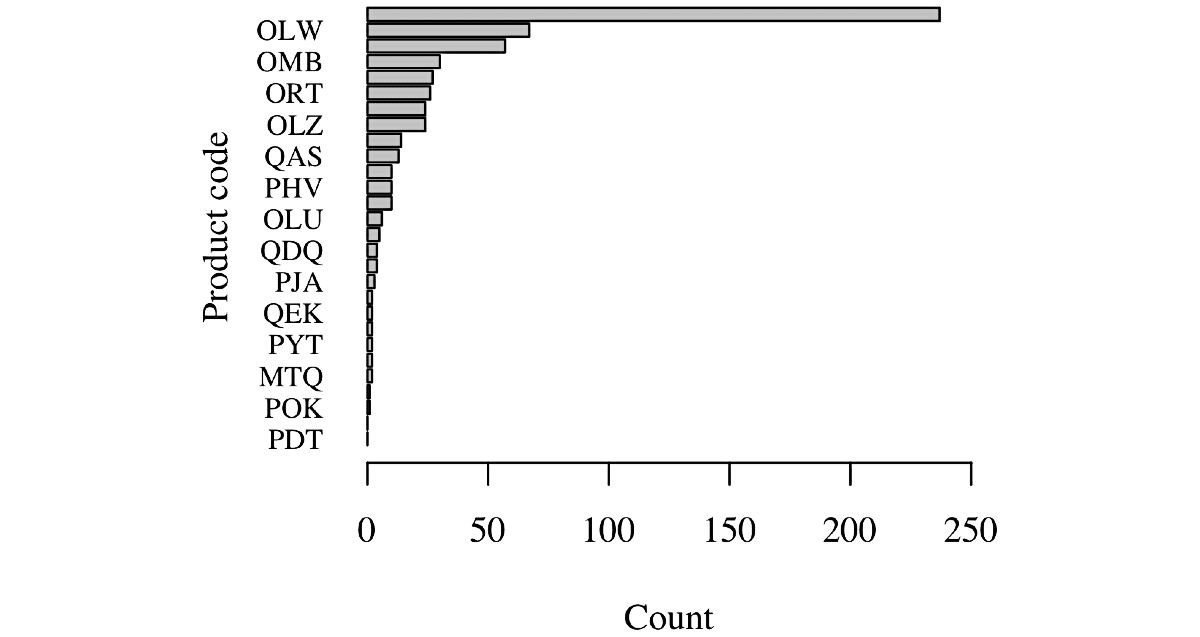
Frequency of software product codes set by the US Food and Drug Administration [13], showing the dominance of index-generating electroencephalograph software (product code OLW) among SaMD registrations.

Index-generating electroencephalograph software (product code OLW [13]) dominates SaMD registrations in the data set (Figure 3), comprising more than one-third of the total devices (187/515, 36.3%).

The pathways to classification are shown in Table 3. The table shows that the majority of devices were submitted through premarket notification, while only 5 devices, comprising <1% of the total devices, followed a 510(k) exemption path.

**Figure 4:**
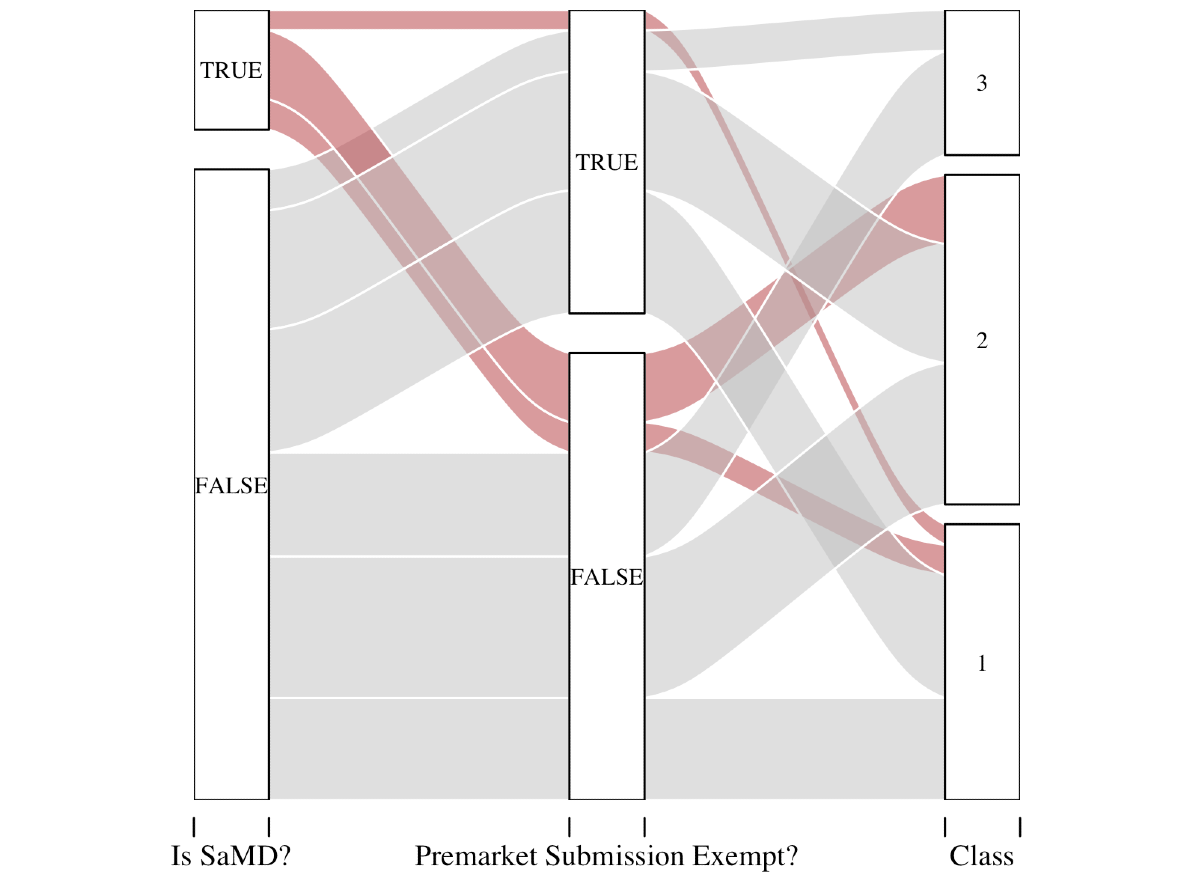
Paths to classification compared between SaMD (red) and non-SaMD (grey) devices. The data are shown in log scale to visualize the distinctions between the paths. The questions are given at the bottom of each column. SaMD: software as a medical device.

The pathway to classification is shown for both SaMD and non-SaMD devices (Figure 4). SaMD, almost without exception, seem to have a more singular path to classification. Their main path for market entry is through premarket notification.

**Table 3:** Pathways to classification for software as a medical device (n=515).

Submission type	Value, n (%)
Premarket notification (510(k))	483 (93.8)
Contact office of device evaluation	22 (4.3)
510(k) exempt	5 (0.97)

### Adverse Events

**Figure 5:**
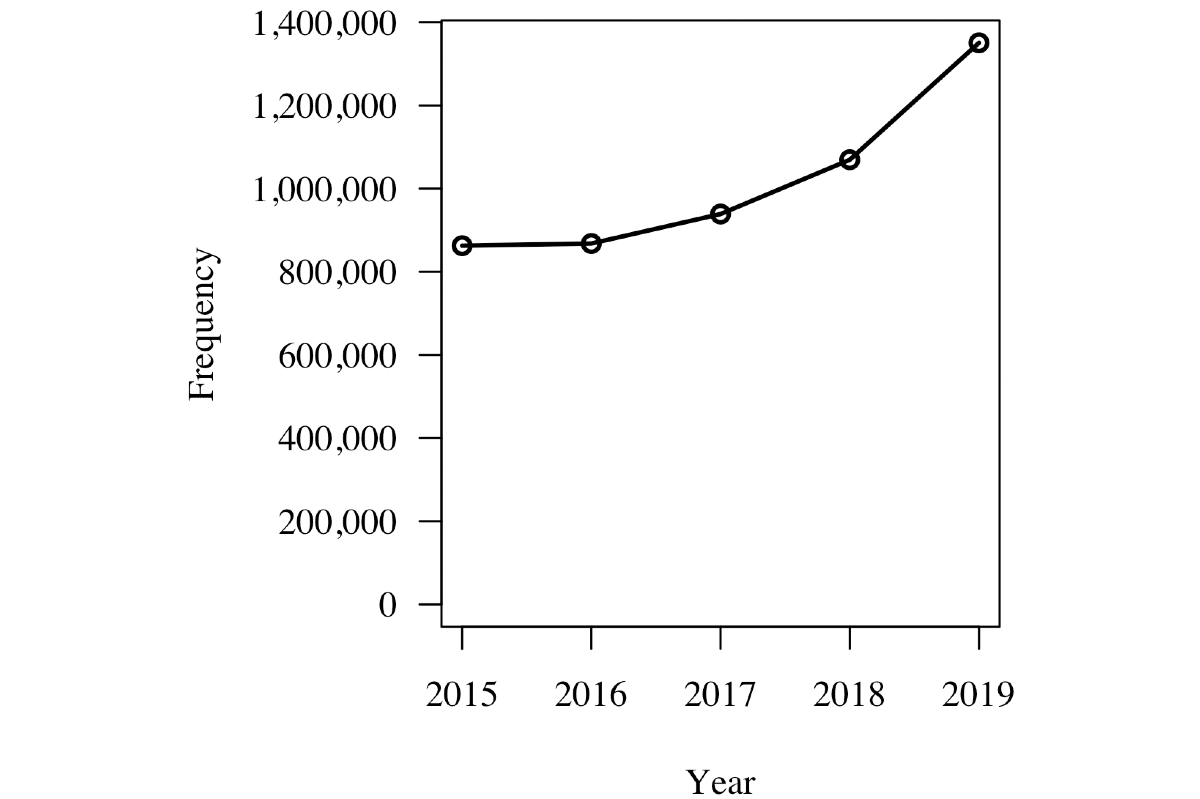
Total adverse events for all medical devices reported in the Manufacturer and User Facility Device Experience database.

**Figure 6:**
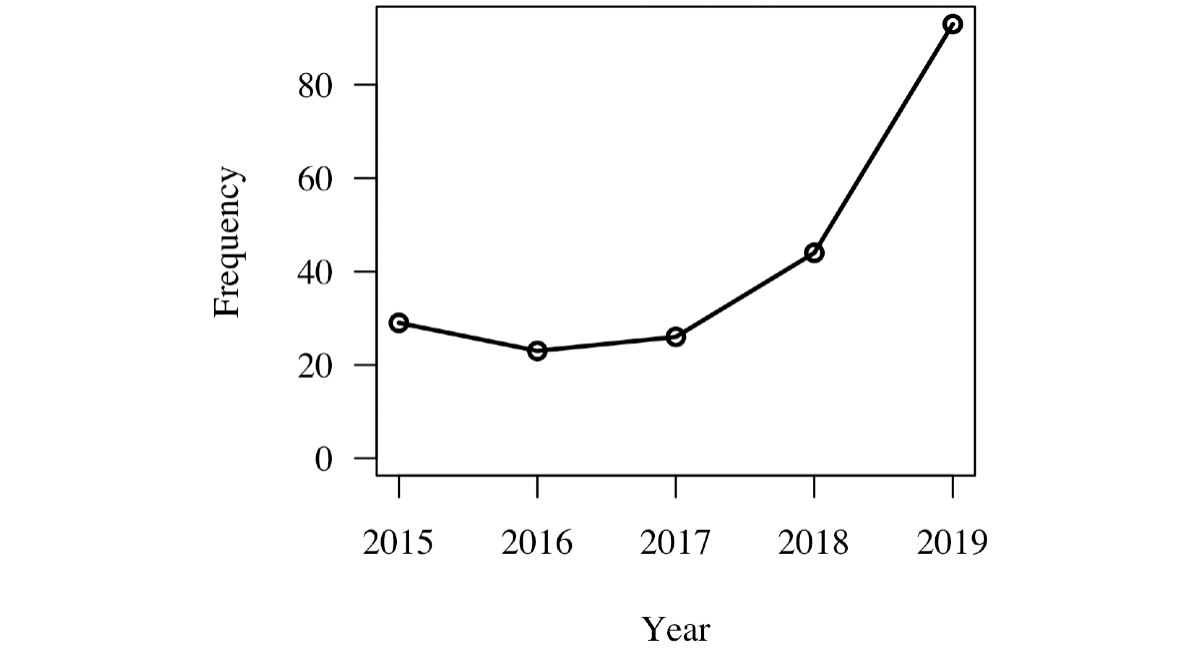
Adverse events for software as a medical device reported in the Manufacturer and User Facility Device Experience database.

For the years from 2015 to 2019, there were 5.1 million reported adverse events in MAUDE for all devices (Figure 5). A subset of the database was examined consisting of only those product codes related to software during the years available for the GUDID. During the same reporting period, 215 adverse events were reported for devices with product codes related to software. This represents a total of 38 manufacturers. This subset does not capture all software-related events but only those related to SaMD (Figure 6). In slightly over half the SaMD cases (21/38, 55%), the device was reported to have been evaluated by the manufacturer. This is in contrast to 37% (1,900,000/5,100,000) of the cases for all reported adverse events.

## Discussion

This work represents a first empirical look at SaMD pathways to market and representation in adverse events based on publicly available data. These findings give a clearer understanding of the nature of SaMD within the regulatory environment. SaMD patterns for entry into market and in adverse events do not seem to deviate from those of medical devices in general. However, the number of new devices entering into the market and adverse events for both types of devices have been rising in the last few years. This rise, however, is rather modest, and it seems that regulations may be an (appropriate) barrier, as not all technologies developed are indeed safe or perform at a suitable level. The number of adverse incidents related to SaMD has also been rising, but at a faster rate than the number of devices. This could indicate that software enters the market earlier than it should, or it may simply identify a tendency toward better reporting of adverse events. This may also explain the higher percentage of adverse event reporting that was found for SaMD manufactures. However, the number of adverse events reported for SaMD is so small compared to the overall number of reported events that any interpretation needs to be carefully considered.

Almost all SaMD requires a 510(k) premarket notification, as demonstrated in Table 3. This indicates that the majority of SaMD is not an exempt product and that manufacturers often aim to enter the market by describing the similarity of their devices to other products that are already available. It has been suggested that although the 510(k) clearing process may offer expediency in bringing devices to market, this may impact the safety of the device. It remains a question for further investigation whether the 510(k) process has a negative impact on the safety of SaMD [15].

There is a noted anomalous spike in the third quarter of 2016 across the data. In that year, the United States passed the 21st Century Cures Act [16], which was aimed at facilitating the acceleration of medical product development by fast-tracking new innovations and advances that could benefit patients. This may have had an effect on the approval of devices. The rise in new device approvals that year may be related to the requirements of this legislation and the reclassification of some devices. It should be noted that GUDID is reliant on device labelers for information and the system allows for bulk uploads, which could help explain this feature in the data. However, no causation data are available to verify this.

Several challenges still remain in developing SaMD. The modern software development pattern frequently uses a form of iterative cycles wherein problems (as well as needed features) are identified and developed within the cyclonic period. However, safety-critical software requires formal verification to determine that it performs as intended and that it can manage identified risks appropriately. Although there is strong evidence that formal verification methods more readily address regulatory compliance, the associated documentation, management, and training costs may not directly contribute to the delivery of customer value [17]. As such, medical device regulations may arguably make it difficult to use modern software development approaches [18]. The FDA has embarked on approaches designed to address the particular issues of software management within health care, such as the use of precertification of SaMD. Lee and Kesselheim [19] highlight that the FDA does not have the resources to validate every single iteration of software. Therefore, if new features are added, they may have certification despite not having any clinical evidence to support claims of treatment or diagnosis. This arguably limits the surveillance that can be conducted by the regulator. This may also have an impact on risk management, which in turn has an effect on the regulatory outcomes for such devices. It is possible that software development itself is not yet optimized for medical devices. Likewise, a question remains as to whether artificial intelligence and machine learning should be distinguished from SaMD, which at the time of publication was an ongoing discussion topic within the FDA [20].

Overall, it seems that SaMD has not yet developed at a different rate from that of other medical devices. Although more research is needed to robustly explain the results reported here, this work does provide useful insight for considering the digital revolution in medicine and how it relates to the market reality.

## Abbreviations

FDA: US Food and Drug Administration
GMDN: Global Medical Device Nomenclature
GUDID: Global Unique Device Identification Database
IMDRF: International Medical Device Regulators Forum
MAUDE: Manufacturer and User Facility Device Experience
PMA: premarket approval
SaMD: software as a medical device

## References

[1] The Lancet Digital Health (2019). A digital (r)evolution: introducing The Lancet Digital Health. Lancet Digit Health.

[2] (2018). Software as a Medical Device (SaMD). US Food and Drug Administration.

[3] Herman W, Devey G (2011). Future trends in medical device technologies: a ten-year forecast. US Food and Drug Administration.

[4] McHugh M, McCaffery F, Casey V (2011). Standalone software as an active medical device.

[5] Tsang L, Kracov D, Mulryne J, Strom L, Perkins N, Dickinson R, Wallace VM, Jones B (2017). The impact of artificial intelligence on medical innovation in the European Union and United States. Intell Prop Tech L J.

[6] 52 Stat. 1040 (Pub. Law 75-717): An act to prohibit the movement in interstate commerce of adulterated and misbranded food, drugs, devices, and cosmetics, and for other purposes. U.S. Law.

[7] (2020). Classify your medical device. US Food and Drug Administration.

[8] Crumpler E, Rudolph H (1997). FDA software policy and regulation of medical device software. Food Drug Law J.

[9] (2013). Software as a Medical Device (SaMD). International Medical Device Regulators Forum.

[10] AccessGUDID. US National Library of Medicine.

[11] Download GUDID data. US National Library of Medicine.

[12] (2020). What is GMDN? GMDN: the standard for naming and grouping medical devices. GMDN Agency.

[13] Product code classification database. US Food and Drug Administration.

[14] MAUDE - Manufacturer and User Facility Device Experience. US Food and Drug Administration.

[15] Institute of Medicine (2011). Medical Devices and the Public's Health: The FDA 510(k) Clearance Process at 35 Years.

[16] Public Law 114–255—Dec. 13, 2016: An act to accelerate the discovery, development, and delivery of 21st century cures, and for other purposes. U.S. Law.

[17] Lin W, Fan X (2009). Software development practice for FDA-compliant medical devices. Proceedings of the Second International Joint Conference on Computational Sciences and Optimization.

[18] McHugh M, McCaffery F, Casey V (2012). Barriers to adopting agile practices when developing medical device software. Software Process Improvement and Capability Determination.

[19] Lee TT, Kesselheim AS (2018). U.S. Food and Drug Administration precertification pilot program for digital health software: weighing the benefits and risks. Ann Intern Med.

[20] (2020). Artificial intelligence and machine learning in software as a medical device. US Food and Drug Administration.

